# Loss of the calorie restriction response protein DEPP1 worsens diet-induced obesity

**DOI:** 10.1210/endocr/bqag075

**Published:** 2026-07-03

**Authors:** Sepideh Sheybani-Deloui, Kripa Shankar, Angie L Bookout, Juan A Rodriguez, Salil Varshney, Omprakash Singh, Connor Lawrence, Jake Tessnow, Aki Uchida, Deepali Gupta, Moyu Lyu, Avi W Burstein, Shota Takemi, Sherri Osborne-Lawrence, Jeffrey M Zigman

**Affiliations:** Department of Internal Medicine, Center for Hypothalamic Research, University of Texas Southwestern Medical Center, Dallas, TX 75390-9030, USA; Department of Internal Medicine, Center for Hypothalamic Research, University of Texas Southwestern Medical Center, Dallas, TX 75390-9030, USA; Department of Internal Medicine, Center for Hypothalamic Research, University of Texas Southwestern Medical Center, Dallas, TX 75390-9030, USA; Department of Internal Medicine, Center for Hypothalamic Research, University of Texas Southwestern Medical Center, Dallas, TX 75390-9030, USA; Department of Internal Medicine, Center for Hypothalamic Research, University of Texas Southwestern Medical Center, Dallas, TX 75390-9030, USA; Department of Internal Medicine, Center for Hypothalamic Research, University of Texas Southwestern Medical Center, Dallas, TX 75390-9030, USA; Department of Internal Medicine, Center for Hypothalamic Research, University of Texas Southwestern Medical Center, Dallas, TX 75390-9030, USA; Department of Internal Medicine, Center for Hypothalamic Research, University of Texas Southwestern Medical Center, Dallas, TX 75390-9030, USA; Department of Internal Medicine, Center for Hypothalamic Research, University of Texas Southwestern Medical Center, Dallas, TX 75390-9030, USA; Department of Internal Medicine, Center for Hypothalamic Research, University of Texas Southwestern Medical Center, Dallas, TX 75390-9030, USA; Department of Internal Medicine, Center for Hypothalamic Research, University of Texas Southwestern Medical Center, Dallas, TX 75390-9030, USA; Department of Internal Medicine, Center for Hypothalamic Research, University of Texas Southwestern Medical Center, Dallas, TX 75390-9030, USA; Department of Internal Medicine, Center for Hypothalamic Research, University of Texas Southwestern Medical Center, Dallas, TX 75390-9030, USA; Department of Internal Medicine, Center for Hypothalamic Research, University of Texas Southwestern Medical Center, Dallas, TX 75390-9030, USA; Department of Internal Medicine, Center for Hypothalamic Research, University of Texas Southwestern Medical Center, Dallas, TX 75390-9030, USA; Division of Endocrinology & Metabolism, Department of Internal Medicine, University of Texas Southwestern Medical Center, Dallas, TX 75390-9030, USA; Peter O’Donnell Jr. Brain Institute, UT Southwestern Medical Center, Dallas, TX 75390-8823, USA; Department of Psychiatry, University of Texas Southwestern Medical Center, Dallas, TX 75390-9070, USA

**Keywords:** calorie restriction, Depp1, diet-induced obesity, ghrelin

## Abstract

The transcriptional landscape of the gastric mucosa in response to opposing nutritional states remains poorly defined. Here, we profiled gastric mucosal gene expression changes induced by diet-induced obesity (DIO) and calorie restriction (CR) in mice and investigated the physiological role of *Decidual Protein Induced by Progesterone 1* (*Depp1*) using newly generated Depp1-knockout (KO) mice. RNA sequencing revealed that DIO elicits a predominantly proinflammatory transcriptional program in the gastric mucosa, whereas CR upregulates genes involved in peptide transport and extracellular matrix organization while downregulating immunity-related pathways. Among CR-induced genes, *Depp1* exhibited the strongest positive correlation with expression of gene encoding the gastric hormone ghrelin. qRT-PCR confirmed enrichment of *Depp1* in gastric ghrelin cells and demonstrated CR-induced upregulation of *Depp1* in additional tissues, including liver, kidney, and pancreas; notably, hepatic induction by CR was absent in ghrelin-KO mice. Despite this association, Depp1-KO mice displayed normal metabolic responses to CR, including preserved glucose homeostasis. In contrast, following 16 weeks of ad libitum high-fat diet feeding, male Depp1-KO mice exhibited greater weight gain, hyperphagia, increased fat and lean mass, and impaired glucose tolerance compared with wild-type littermates. These phenotypes were accompanied by selective hepatic gene expression changes affecting *Pgc1a*, *Pck1*, and *Igf1*. Collectively, these findings identify *Depp1* as a CR-induced, ghrelin-associated gene that influences hepatic transcriptional responses yet is dispensable for short-term adaptation to CR, while also implicating *Depp1* as a protective factor against metabolic dysfunction during DIO through mechanisms that remain to be defined.

Energy homeostasis is a tightly regulated process governed by dynamic interactions between the central nervous system and a diverse array of peripheral tissues. Beyond its traditional digestive roles, the gastrointestinal (GI) tract has emerged as a pivotal endocrine organ that actively contributes to systemic energy balance (1-3). In this capacity, the GI tract functions as a dynamic nutrient sensor, responding to ingested food by secreting peptide hormones that coordinate whole-body metabolic responses (1, 2). These include glucagon-like peptide-1, secreted by enteroendocrine L cells of the distal small intestine and colon, and glucose-dependent insulinotropic polypeptide, secreted by enteroendocrine K cells of the proximal small intestine, the receptor pathways of which are harnessed by the most efficacious anti-obesity pharmacotherapies currently available.

Within the GI tract, the stomach plays a particularly important role in regulating energy balance (4-6). In addition to its mechanical and digestive functions, the stomach contributes through nutrient sensing and hormone secretion, most notably via the acylated hormone ghrelin (7-9). Ghrelin is produced by enteroendocrine ghrelin cells located primarily in the gastric mucosa and exerts myriad effects on energy balance, including stimulating appetite, reducing energy expenditure, promoting gastric motility, enhancing gastric acid secretion, and raising blood glucose (10-15). These cells, which comprise ∼0.3% to 1% of gastric mucosal cells, regulate ghrelin release from secretory granules in response to sympathetic nervous system input, circulating factors, and mechanical stretch (4, 16-18). The stomach also produces additional hormones and paracrine signals through distinct enteroendocrine cell types, including gastrin (G cells), somatostatin (D cells), serotonin (enterochromaffin cells), and histamine (enterochromaffin-like cells) (19-22).

Calorie restriction (CR) and diet-induced obesity (DIO) represent opposing nutritional states that drive profound and divergent transcriptional and physiological adaptations across peripheral tissues. Calorie restriction, defined as sustained reduction in energy intake, enhances insulin sensitivity, promotes mitochondrial biogenesis, and suppresses inflammatory signaling, thereby supporting metabolic efficiency and cellular resilience (23-25). In contrast, chronic consumption of energy-dense diets [eg, high-fat diet (HFD)], drives DIO, which in turn often leads to insulin resistance, hepatic steatosis, and systemic inflammation (26-28). Although these adaptations have been extensively characterized in organs such as the liver, adipose tissue, hypothalamus, and intestine (29-34), far less is known about their effects on the gastric mucosa.

Prior work has shown that gastric ghrelin cells respond dynamically to these nutritional states. Calorie restriction stimulates ghrelin secretion via sympathetic activation of ghrelin cell β_1_-adrenergic receptors and engagement of the Notch-FOXO1 pathway, whereas DIO suppresses ghrelin release mainly through insulin receptor–mediated signaling (17, 35, 36). However, beyond these ghrelin cell-specific adaptations, the broader transcriptional responses of the gastric mucosa to CR and DIO remain largely undefined. Elucidating these responses is essential for understanding how gastric nutrient sensing contributes to systemic metabolic regulation during states of energy deficit and excess.

To address this gap, we characterized the transcriptional responses of the gastric mucosa to CR and DIO in mice. Bulk RNA-sequencing (RNA-seq) was used to compare gene expression across lean, CR, and DIO conditions, and differentially expressed genes (DEGs) were interrogated using single-cell RNA-seq (scRNA-seq) datasets to assess co-expression with ghrelin. Given the marked induction of *Depp1* (*Decidual Protein Induced by Progesterone 1*) in the gastric mucosa and other tissues during CR, we generated a Depp1-knockout (KO) mouse line to evaluate the physiological consequences of *Depp1* loss in both CR and DIO contexts. This integrated approach identified DEPP1 as a nutrient-responsive gene and defined its contribution to systemic metabolic adaptation across contrasting nutritional states.

## Materials and methods

### Animal care

Mice were housed at 21.5 °C to 22.5 °C with a 12-hour light/12-hour dark cycle with free access to standard chow (Teklad Global 16% Protein Rodent Diet, 2916; Envigo, Indianapolis, IN, United States) and water, unless otherwise noted. All experiments were approved by the UT Southwestern Medical Center Institutional Animal Care and Use Committee. Ghrelin-knockout (GKO) and wild-type (WT) littermate mice were generated by crossing GKO (line GKO1) heterozygotes on a C57BL/6N background, as previously reported (37, 38). The *Ghrl^Cre^* mouse line was generated as previously described and maintained on C57BL/6N background (39). For reporter studies, *Ghrl^Cre^* mice were crossed to *Rosa26-lox-STOP-lox-tdTomato* reporter mice [B6.Cg-Gt(ROSA)26Sor^tm14(CAG-tdTomato)Hze/J^; The Jackson Laboratory; RRID:IMSR_JAX:007914] to generate *Ghrl^Cre^;tdTomato* mice.

### Generation of Depp1-KO mice

Depp1-KO mice were generated using CRISPR/Cas9 technology via microinjection of Cas9 mRNA, 2 short-guide RNAs (sgRNAs), and 2 donor oligonucleotides containing loxP sites flanked by homology arms into C57BL/6N zygotes at the UT Southwestern Transgenic Core Facility; only deleted *Depp1* alleles were recovered. The Cas9-mediated double-strand breaks were directed by guide RNAs targeting the *Depp1* locus. The 5′ sgRNA was designed to bind the genomic site 228 bp downstream of Exon 1 and 596 bp upstream of Exon 2 on the non-coding strand (5′-TAGGGTTATAAACATCCΔCAC-3′; Cas9 cut site indicated by Δ). The 3′ sgRNA was designed to bind 116 bp downstream of the end of Exon 2, which is 648 bp downstream of the STOP codon (5′-CTTCAGGCATATATTTCΔCAG-3′).

One line (DKOe) was selected for further study. This line harbors a deletion predicted to ablate DEPP1 protein production. Loss of *Depp1* expression was confirmed by RT-qPCR in multiple tissues. The WT allele was detected using m817 (5′-CCACTTAATGACTTAGAGACTG-3′) and m800 (5′-GTGTCCACCACAGGAGAGGA-3′), producing a 224 bp product. The KO allele is detected using m801 (5′-CTGACTACTGGATAGAGAGCA-3′) and m800, yielding a 299 bp product.

### Long-term feeding studies

Three-week-old male C57BL/6N mice were purchased from Charles River Laboratories (RRID:IMSR_CRL:027; Wilmington, MA, United States). Mice were singly housed and fed an ad libitum standard chow diet (2916, Teklad) for 7 days. At 4 weeks of age, mice were randomly assigned to one of these groups (*n* = 7-10/group): (1) ad libitum chow, (2) ad libitum Western-style HFD (Envigo Teklad TD88137; 4.5 kcal/g, 42% kcal from fat), or (3) 60% CR (see below).

For feeding studies in Depp1-KO and WT littermates, 4-week-old male and female mice were individually housed and randomly assigned to the following groups: (1) Ad libitum HFD (TD88137, Envigo Teklad) for 16 weeks or (2) 60% CR. Body weight and food intake were measured weekly. Body composition analysis was performed monthly using an EchoMRI-100 apparatus (EchoMRI LLC, Houston, TX, United States).

Calorie restriction was performed using mice that initially had been individually housed for 1 week with ad libitum access to this standard chow diet: 5L0D (PicoLab^®^ Laboratory Rodent Diet, LabDiet, St Louis, MO, United States). During this acclimatization period, daily food intake was measured for the last 5 days to determine the mean usual daily caloric intake for each mouse. Mice were then provided access to 40% of their usual daily caloric intake in the form of this same diet once daily at 5:30 Pm (30 minutes prior to lights-off) for 7 or 9 days (as indicated in the text). Percentage body fat mass was determined immediately prior to beginning the CR protocol and again at the end of the study. Body weight and blood glucose were measured daily just prior to delivery of the food ration. On the final day of the study, mice were euthanized just prior to food delivery, and trunk blood was collected for downstream analyses. As mice rapidly consumed the provided food, they experienced an acute fast of ∼22 to 22.5 hours at the time of sample collection on top of the prolonged CR (“acute-on-chronic” CR).

### Glucose and insulin tolerance tests

For glucose tolerance testing (GTT), 16-week-old HFD-fed male and female Depp1-KO and WT littermates were fasted for 6 hours (beginning at ∼7:00 Am). Glucose (2 g/kg BW, Sigma-Aldrich) was administered by oral gavage. Blood glucose was measured from nicked tails using a Contour Next EZ monitoring system (Bayer) at −5 (fasting), 30, 60, 90, and 120 minutes post-gavage. For insulin tolerance testing (ITT), mice were fasted for 6 hours before testing. Insulin (Humulin R; Eli Lilly and Company, Indianapolis, IN, United States) was diluted in sterile saline (0.9%) and injected i.p. (1 U/kg BW). Blood glucose was measured at 5, 30, 60, 90, and 120 minutes postinjection.

### Ghrelin administration studies

Seven-week-old male C57BL/6N mice (RRID:IMSR_CRL:027; Charles River Laboratories) were administered ghrelin [2 mg/kg body weight, s.c.; Ghrelin (rat), SP-GHRL, Innovagen AB, Lund, Sweden] or an equivalent volume of sterile saline as control. Food intake was measured for 2 hours postinjection. For gene expression analyses, gastric mucosal cells (GMCs) and liver tissues were collected and stored in RNALater (AM7020; Thermo Fisher Scientific, Waltham, MA, United States) 2 hours postinjection.

### Plasma hormone analysis

Blood samples were collected following quick decapitation into prechilled EDTA-coated microtubes containing the protease inhibitor p-hydroxymercuribenzoic acid (Sigma-Aldrich; final concentration 1 mM). Samples were centrifuged immediately at 4 °C at 1500 × *g* for 15 minutes. For stabilization of the acyl-group on ghrelin, HCl was added (1:10) to the plasma to achieve a final concentration of 0.1N. Samples were stored at −80 °C. Ghrelin was measured using a Rat/Mouse Ghrelin ELISA Kit (EZRGRA-MilliporeSigma, Burlington, MA, United States; RRID:AB_2801454).

### Histology

Liver and left epididymal white adipose tissue (eWAT) were collected and fixed in 10% formalin at 4 °C for 2 to 4 days. Paraffin processing and embedding were performed at the UT Southwestern HistoPathology Core. Serial 5 µm sections were cut on a Leica RM2255 rotary microtome and stained with H&E on a Sakura Prisma Plus robotic stainer. Images were acquired using a Leica DM6 B microscope with a Leica DFC 9000 GT camera. Three to four sections per sample (separated by at least 100 µm) were analyzed.

### Preparation of gastric mucosal cells

Mice were anesthetized, and stomachs were tied off at both ends with surgical suture, excised, and placed in ice-cold PBS. Stomachs were incised at the nonglandular portion to remove luminal contents, inverted inside-out by passing the distal stomach through the incision, inflated, and briefly placed in ice-cold glucose-free DMEM. Residual digesta adherent to the mucosa was gently removed using soft paper pads. The inverted, inflated stomachs were incubated for 1.5 hours at 37 °C in 35 U dispase II/3 mL PBS, after which the exposed gastric mucosa was scraped off and collected. Gastric mucosal cells were dispersed, centrifuged, and digested with 0.25% trypsin-EDTA for 5 minutes. The suspension was filtered through a 100 µm cell strainer, centrifuged, resuspended in Fluorescence-Activated Cell Sorting (FACS) buffer, and filtered again through a 30 µm cell strainer before downstream processing.

For ghrelin-cell enrichment studies, 8-week-old male *Ghrl*^Cre^;*tdTomato* reporter mice were used (*n* = 3). For these studies, cells were sorted on a BD FACSAria III Cell Sorter to isolate Tom+ (ghrelin) and Tom− (non-ghrelin) populations. Cells were collected directly into extraction buffer from the Arcturus™ PicoPure™ RNA Isolation Kit (KIT0204; Thermo Fisher Scientific) and stored at −80 °C.

### Total RNA isolation and bulk RNA-seq

Total RNA from GMC, pancreas, kidney, and liver tissues was extracted using RNA STAT-60 (AMSBio) and purified using the RNeasy Mini Kit (74104; QIAGEN, Germantown, MD, United States). RNA quality for bulk RNA-seq was assessed using Qubit and Agilent 2100 Bioanalyzer at UT Southwestern Microarray and Immune Phenotyping Core Facility RNA from 5 CR, 5 DIO, and 4 Lean mice (RIN ≥ 9) was used for library construction and pair-end sequencing (2 × 150 bp) on an Illumina NovaSeq X Plus (Novogene Corporation Inc., Sacramento, CA, United States).

### Bulk RNA-seq analysis

After performing data filtering and quality control of the raw RNA-seq data, we aligned the filtered reads to the mouse genome (GRCm39 Ensembl release 104) using the STAR software (RRID:SCR_015899) (40). Gene abundance was quantified using featureCounts (Subread package) to generate raw read counts. PCA was performed to assess sample variance; 1 sample from the lean group and 1 sample from the DIO group were identified as outliers and removed prior to further analysis. Differentially expressed genes were identified using DESeq2 R package (RRID:SCR_015687) (41); genes with a |log2 (fold-change)| ≥ 1 and a Benjamini–Hochberg–adjusted *P*-value (false discovery rate) of ≤.05 were defined as DEGs unless otherwise stated. Gene ontology (GO) term enrichment analysis was done by Clusterprofiler R package using GO term biological processes database. Venn diagrams, volcano plot of DEGs, and dot plots of enriched GO terms were generated using the R packages, EnhancedVolcano and ggplot2 (42).

### Single-cell RNA-seq analysis

Processed and annotated data were downloaded from the Gene Expression Omnibus (accession number: GSE134520), and gene expression was visualized using R package Seurat (RRID:SCR 016341). Cell clustering and cell type annotations were derived from the original publication. Normalized expression data for *GHRL* and selected genes were extracted, and Pearson correlation coefficients were calculated using log-normalized expression values.

### Quantitative reverse-transcriptase PCR

Total RNA was isolated using RNA STAT-60 (CS-110; AMSBIO, Cambridge, United Kingdom) and quantified by Nanodrop. cDNA was synthesized using SuperScript III (18080044; Invitrogen/Thermo Fisher Scientific). Quantitative PCR was performed using the QuantStudio 5 System. The following SYBR chemistry primers were used: *Depp1* (F: 5′-GCTAGAGACCCGGCTGTGACT-3′, R: 5′-GGCACGGGCAGCAGAA-3′), *Pgc1a* (F: 5′-TATGGAGTGACATAGAGTGTGCT −3′, R: 5′-CCACTTCAATCCACCCAGAAAG −3′), *Igf1* (F: 5′-GGCATTGTGGATGAGTGTTG-3′, R: 5′-TCTCCTTTGCAGCTTCGTTT-3′), *Fgf21* (F: 5′-CCTCTAGGTTTCTTTGCCAACAG-3′, R: 5′-AAGCTGCAGGCCTCAGGAT-3′), *Leap2* (F: 5′-GCTGCTGGGTCAGGTCAATAG-3′, R: 5′-CCGGGATCTCTTTGCTGAAC-3′), and *Pck1* (F: 5′-TGACAACTGTTGGCTGGCTC-3′, R: 5′-GACATACATGGTGCGGCCTT-3′). mRNA levels were normalized using the ΔΔCt method. Unless otherwise stated, gene expression was normalized to *18s* (Mm04277571_s1; Thermo Fisher Scientific). *B2m* (SYBR F: 5′-TTCTGGTGCTTGTCTCACTGA-3′, R: 5′-CAGTATGTTCGGCTTCCCATT-3′) was used as the reference gene for selected experiments when it showed greater stability across the experimental conditions tested, as indicated in the corresponding figure legends. Each reference gene was validated within its respective experiment for stability across conditions.

### Statistical analysis

Apart from those for the bulk RNA-seq data, statistical analyses and graph preparations were performed using GraphPad Prism 10.4.1, as indicated in the figure legends. Cohorts were analyzed separately by sex where indicated. The data are presented as mean ± SEM. Post hoc analyses were performed when appropriate (eg, if a 1-way ANOVA showed a significant effect of the factor or if a 2-way ANOVA showed a significant interaction between factors), as indicated in the figure legends. Outliers were detected using the ROUT test and removed. *P*-values <.05 were considered statistically significant, while *P*-values ≥.05 and <.1 were considered evidence of a statistical trend.

## Results

### Dietary condition-specific gene expression in gastric mucosa

**Figure 1:**
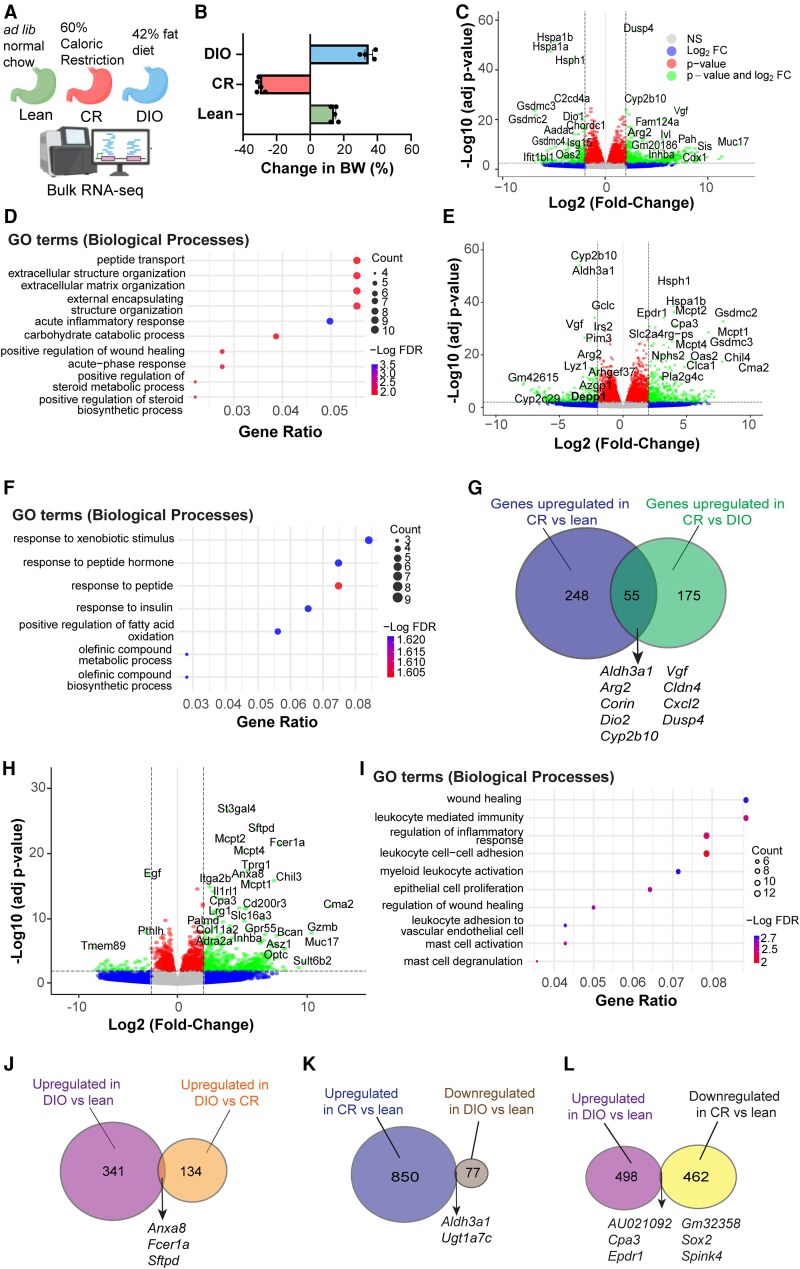
Transcriptomic analysis reveals distinct gene expression profiles in lean, DIO, and CR mice. (A) Schematic representation of the experimental strategy for bulk RNA-seq in Lean, CR, and DIO mice. Created with BioRender.com. (B) Bar graph depicting the percentage change in body weight (BW) for Lean, CR, and DIO groups. (C) Volcano plot illustrating the DEGs in the CR group compared with the Lean group. (D) Dot plot showing the GO terms (Biological Processes) enriched in genes upregulated in the CR group vs the Lean group. (E) Volcano plot illustrating the DEGs in the CR group compared with the DIO group. (F) Dot plot showing the GO terms (Biological Processes) enriched in genes upregulated in the CR group vs the DIO group. (G) Venn diagram displaying the overlap of upregulated genes between the CR vs Lean and CR vs DIO comparisons, with a selection of representative genes listed. (H) Volcano plot illustrating the DEGs in the DIO group compared with the Lean group. (I) Dot plot showing the GO terms (Biological Processes) enriched in genes upregulated in the DIO group vs the Lean group. (J) Venn diagram displaying the overlap of upregulated genes between the DIO vs Lean and DIO vs CR comparisons, with a selection of representative genes listed. (K) Venn diagram showing the overlap between genes upregulated in CR vs Lean and genes downregulated in DIO vs Lean, with representative genes listed. (L) Venn diagram showing the overlap between genes upregulated in DIO vs Lean and genes downregulated in CR vs Lean, with representative genes listed. In dot plots, gene ratio represents the proportion of significantly DEGs associated with each GO term, dot size indicates the number of DEGs associated with the term (count), and color indicates enrichment significance as −log10(false discovery rate, FDR).

To identify novel or understudied gastric mucosal genes that may enable the body to sense and adapt to opposing nutritional extremes, we used bulk RNA-seq as a discovery pipeline to profile gene expression across 3 conditions. Specifically, we examined GMC isolated from mice fed standard chow ad libitum for 4 weeks (lean; *n* = 3), mice subjected to a 7-day acute-on-chronic CR protocol modeling starvation (CR; *n* = 5), and mice fed a 42% HFD for 4 weeks (DIO; *n* = 4; Fig. 1A; see Materials and Methods for details). Over the course of 4 weeks, the lean group experienced a 14.2 ± 0. 8% BW gain, while the DIO group experienced a 34.8 ± 2.0% BW gain. The CR group experienced a 29.9 ± 0.8% BW loss over 7 days (Fig. 1B).

We first compared the CR and lean groups to establish a baseline of pathways and cellular processes specifically responsive to energy restriction. Using a Benjamini–Hochberg–adjusted *P* < .01 and log2 (fold-change) ≥2 as thresholds for statistical and biological significance, we identified 303 upregulated and 119 downregulated genes [Fig. 1C, Table S1 (43)]. Gene ontology–term enrichment analysis indicated that CR-induced genes were associated with pathways related to peptide transport and extracellular matrix organization, whereas genes involved in innate immune signaling and host defense were suppressed [Fig. 1D; Tables S2 and S3 (43)].

To refine this dataset and identify transcripts associated with opposing nutritional states, we next compared the CR and DIO groups directly. Using the same thresholds, this comparison identified 230 genes upregulated during CR, with enrichment for biological pathways related to peptide and hormone response, as well as insulin signaling (*Vgf, Irs2, Egr1, Klf15, Nr4a1, Sgk1*, and *Obp2a*) [Fig. 1E and 1F, Tables S4 and S5 (43)]. To distinguish genes selectively responsive to CR, rather than general dietary changes, we further examined the overlap between genes upregulated in CR relative to both lean and DIO groups. This analysis identified 55 overlapping DEGs, several of which are implicated in mucosal function [Fig. 1G; Table 1; full list in Table S6 (43)]. Because this overlap-based approach provides a stringent CR-associated signature but may miss biologically relevant genes that distinguish opposing nutritional states, we also interrogated the full CR vs DIO comparison for candidates suitable for downstream analysis. Using a standard significance threshold (adjusted *P* < .05), this analysis identified Decidual Protein Induced by Progesterone 1 [*Depp1*; also known as fasting-induced gene (*Fig*)] (53), which was upregulated 3-fold in CR vs DIO (adjusted *P* = .015; Fig. 1E).

**Table 1:** Functional annotation of key common genes upregulated by calorie restriction in the gastric mucosa relative to lean and DIO

Gene symbol	Gene name	Function (general and/or gastric mucosa related)
*Aldh3a1*	Aldehyde Dehydrogenase 3 Family, Member A1	Detoxification: ALDH3A1 is an enzyme that detoxifies aldehydes. In the stomach, this could be important for protecting the mucosa from ingested toxic aldehydes or those produced during metabolism. It plays a role in cellular defense against oxidative stress (44).
*Arg2*	Arginase 2	Arginine metabolism: ARG2 is an enzyme that catalyzes the hydrolysis of arginine to ornithine and urea. While primarily known for its role in the liver's urea cycle, extrahepatic ARG2 (including in the stomach) can influence nitric oxide (NO) production. May be involved in gastric mucosal defense and repair (45).
*Cldn4*	Claudin 4	Tight junction formation: CLDN4 maintains barrier function of the gastric mucosa (46).
*Corin*	Corin	Natriuretic peptide processing: May play a role in local fluid/electrolyte regulation in the GI tract (47).
*Cxcl2*	Chemokine (C-X-C Motif) Ligand 2	Chemotaxis and inflammation: CXCL2 is upregulated during inflammation in the stomach (48).
*Dio2*	Deiodinase, Iodothyronine, Type II	Thyroid hormone activation: May influence gastric acid secretion and mucosal cell turnover (49).
*Dusp4*	Dual Specificity Phosphatase 4	Mitogen-Activated Protein kinase (MAPK) regulation: DUSP4 could regulate mucosal cell turnover and responses to stress (50).
*Vgf*	VGF Nerve Growth Factor Inducible	Neuropeptide precursor: In the stomach, VGF may regulate motility, secretion, and mucosal protection. Neuroprotective and anti-inflammatory effects (51, 52).

We next assessed transcriptional changes induced by dietary fat exposure. Compared with lean controls, DIO mice exhibited predominantly upregulated DEGs, with 344 genes enriched for immune-related pathways, including inflammatory responses, leukocyte-mediated immunity, and myeloid leukocyte activation, among others [Fig. 1H and 1I, Tables S7 and S8 (43)], consistent with the proinflammatory effects of high-fat feeding. Although the DEG profiles of DIO vs lean and DIO vs CR comparisons were largely distinct, inflammatory and immune processes were commonly enriched GO terms in both comparisons. Additional overlap and reciprocal-regulation analyses identified a limited set of diet-specific and oppositely regulated transcripts, which are summarized in Fig. 1J-1L.

### Identification of ghrelin cell genes differentially regulated by CR and high-fat feeding

Having identified a highly dynamic, diet-responsive set of transcripts in the gastric mucosa, we next sought to resolve their specific cellular localization. Given the gastric hormone ghrelin's key role in energy homeostasis and its known responsiveness to both CR and DIO, we examined whether any of the identified DEGs localize to this critical enteroendocrine population. To address this, we employed 2 complementary approaches.

**Figure 2:**
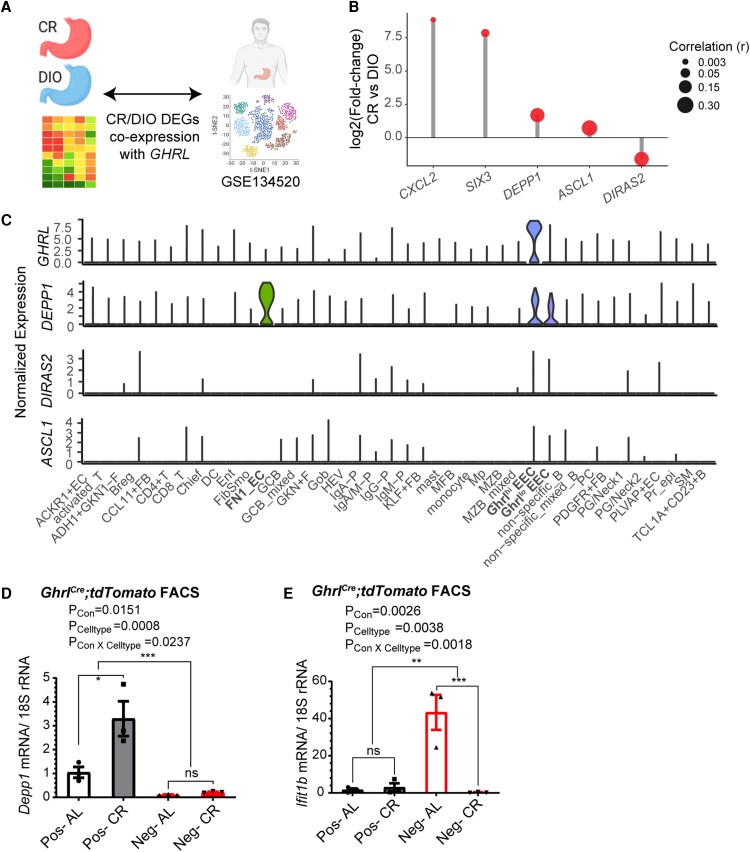
Correlation of differentially expressed genes with ghrelin and validation in sorted cells. (A) Schematic outlining the analysis strategy to correlate DEGs identified in Mouse CR vs DIO bulk RNA-seq with *GHRL* expression in a human stomach single-cell RNA-seq dataset (GSE134520). Created with BioRender.com. (B) Lollipop chart summarizing the correlation (Pearson *r*-value) of selected top DEGs (*ASCL1*, *DIRAS2*, *DEPP1*, *SIX3*, *CXCL2*) with *GHRL* expression in the single-cell dataset. *Y*-axis indicates log2(fold-change) and circle size represents the magnitude of the Pearson *r*-value. (C) Stacked violin plots illustrating the normalized expression distribution of *GHRL*, *DEPP1*, *DIRAS2*, and *ASCL1* across various annotated cell clusters in the gastric scRNA-seq dataset. Note the co-expression patterns in enteroendocrine cells (EECs). FN1_EC denotes fibronectin 1–positive endothelial cells. (D) qPCR analysis of *Depp1* mRNA expression in FACS-sorted ghrelin-positive (Pos) and ghrelin-negative (Neg) gastric cells from *Ghrl^Cre^;tdTomato* mice fed ad libitum (AL) or subjected to CR. (E) qPCR analysis of *Ifit1b* mRNA expression in the same FACS-sorted populations (Pos/Neg, AL/CR). All expression values were normalized to 18S rRNA. Statistical significance was determined by 2-way ANOVA (*P*-values for condition, cell type, and interaction are shown). Pairwise comparisons were performed using Tukey multiple comparisons test. Significant pairwise differences are indicated by asterisks (*P < .05, **P < .01, ***P < .001) and “ns” denotes not significant.

First, we analyzed a publicly available single-cell transcriptomic dataset of the human stomach (54) to evaluate co-expression relationships between *GHRL* (which encodes ghrelin) and the DEGs identified under CR and DIO conditions [Fig. 2A; Fig. S1A (43)]. Pearson correlation analysis revealed *DEPP1* and several other genes, including *CXCL2* (encoding C-X-C motif chemokine ligand 2)*, SIX3* (encoding SIX homeobox 3 transcription factor), *ASCL1* (encoding a Basic helix-loop-helix transcription factor), and *DIRAS2* (encoding a member of RAS superfamily of monomeric GTPases) positively correlated with *GHRL* (r > 0) [Table S9 (43)]. To prioritize functionally relevant candidates, we stratified these genes by log2 (fold-change) magnitude, identifying *CXCL2*, *SIX3*, and *DEPP1*, as robustly upregulated under CR [log2 (fold-change) > 1.5; Fig. 2B]. Within this subset of 3 robustly upregulated genes, *DEPP1* distinguished itself by exhibiting the highest correlation with *GHRL* (Fig. 2B). UMAP visualization further revealed that *DEPP1* expression is enriched within enteroendocrine clusters that co-express *GHRL*, although is not exclusive to this population [Fig. S1B-S1D (43)]. In addition, among the genes with positive correlation with *GHRL*, *DEPP1* showed the highest normalized expression within both *GHRL^hi^* and *GHRL^lo^* enteroendocrine cell clusters (those with high and low *GHRL* expression, respectively; Fig. 2C). These results highlight *DEPP1* as a promising candidate mediator of energy state-dependent regulation in ghrelin cells.

Second, to directly assess *Depp1* expression in ghrelin cells, we submitted GMC from *Ghrl^Cre^;tdTomato* reporter mice to FACS, allowing us to separate ghrelin-positive cells from ghrelin-negative cells. Consistent with the first approach, *Depp1* expression was upregulated in ghrelin-positive cells from CR mice compared with ad libitum standard chow-fed controls (by 114.6%; Fig. 2D), with no significant CR-induced change in ghrelin-negative cells. Conversely, *IFit1b* (interferon-induced protein with tetratricopeptide repeats 1B; involved in innate antiviral immunity), identified as downregulated by CR in bulk RNA-seq of the gastric mucosa, was significantly decreased by CR in ghrelin-negative cells (*P* < .005), but unchanged in ghrelin-positive cells (Fig. 2E).

Together, these complementary analyses demonstrate that within the gastric mucosa, *Depp1* is enriched in ghrelin cells where it is upregulated during CR, supporting a potential role for DEPP1 in modulating ghrelin cell activity and energy state–dependent signaling.

### *Depp1* expression is regulated by calorie restriction in several tissues and by ghrelin in the liver

**Figure 3:**
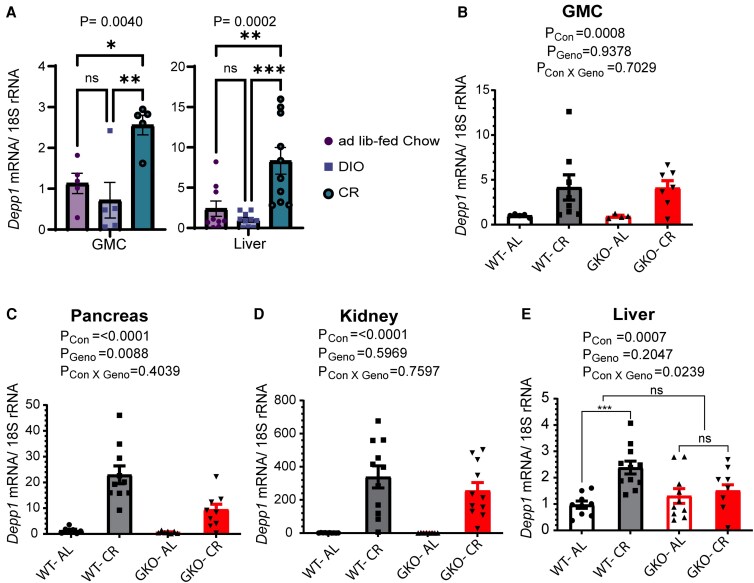
Regulation of *Depp1* expression by diet and ghrelin deficiency. (A) Relative mRNA expression of *Depp1* normalized to *18S* rRNA in the gastric mucosa (GMC) and liver of male mice under ad libitum–fed chow, DIO, and CR conditions. (B) Relative *Depp1* expression normalized to *18S* rRNA in the gastric mucosa (GMC) of WT and GKO mice under ad libitum–fed (AL) and calorie-restricted (CR) conditions. (C) Relative *Depp1* expression in the pancreas of WT and GKO mice under AL and CR conditions. (D) Relative *Depp1* expression in the kidney of WT and GKO mice under AL and CR conditions. (E) Relative *Depp1* expression in the liver of WT and GKO mice under AL and CR conditions. For (A), statistical significance was determined by 1-way ANOVA. For (B-E), statistical significance was determined by 2-way ANOVA; *P*-values for condition (*P*_Con_), genotype (*P*_Geno_), and interaction (*P*_Con×Geno_) are displayed above each graph. Pairwise comparisons were performed using Tukey multiple comparisons test. Significant pairwise differences are indicated by asterisks (*P < .05, **P < .01, ***P < .001) and “ns” denotes not significant. Fold changes reflect relative differences within each tissue and should not be directly compared in magnitude across tissues due to differences in baseline expression levels.

To determine whether CR-induced *Depp1* regulation extends beyond the gastric mucosa, we examined *Depp1* expression in liver from male mice exposed to ad libitum chow, DIO, or CR conditions. Like the response observed in GMC, hepatic *Depp1* expression was significantly increased by CR compared with both ad libitum chow-fed and DIO mice (Fig. 3A). In contrast to males, CR did not significantly alter *Depp1* expression in GMC or liver from female mice [Fig. S2A and S2B (43)]. This was not due to a blunted physiological response to CR, as female and male mice exhibited comparable body weight loss during the CR protocol [Fig. S2C (43)].

We next tested whether CR-induced *Depp1* expression depends on the typical CR-induced rise in endogenous ghrelin by comparing WT and GKO mice under ad libitum–fed and CR conditions across several tissues. Calorie restriction significantly increased *Depp1* expression in GMC, pancreas, kidney, and liver (Fig. 3B-3E). In pancreas, there was also a significant main effect of genotype, with lower overall *Depp1* expression in GKO mice compared with WT mice (Fig. 3C). However, only liver showed a significant condition-by-genotype interaction, with CR inducing *Depp1* expression in WT mice but not in GKO mice (Fig. 3E). These findings indicate that *Depp1* upregulation by CR occurs in several tissues but its dependence on endogenous ghrelin is tissue-specific and most evident in the liver.

To determine whether acute ghrelin administration is sufficient to induce *Depp1* expression, we treated ad libitum standard chow-fed mice with a single dose of ghrelin (2 mg/kg) and measured *Depp1* mRNA levels in GMC and liver. While ghrelin administration significantly increased food intake, it did not alter *Depp1* expression in either tissue [Fig. S3A-S3C (43)], indicating that acute ghrelin exposure alone, under ad libitum–fed condition, is insufficient to recapitulate the effects of CR on *Depp1* expression.

### *Depp1* deletion does not affect metabolic adaptations to calorie restriction

**Figure 4:**
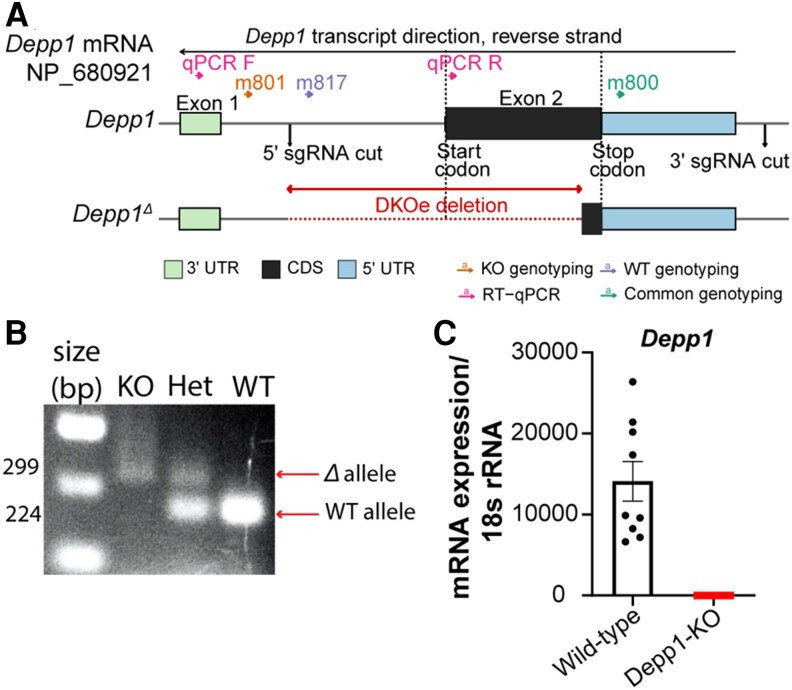
Generation of Depp1-KO mice and validation. (A) Schematic of the mouse *Depp1* locus and the CRISPR/Cas9-generated DKOe allele. *Depp1* is encoded on the reverse strand, with exon structure, untranslated regions (UTRs), coding sequence (CDS), start codon, and stop codon indicated. The DKOe allele (*Depp1^Δ^*) contains a deletion spanning positions 2120-3274, removing the start codon and part of the coding sequence within Exon 2. Locations of sgRNA cut sites, genotyping primers (m817, m800, and m801), and RT-qPCR primers are shown. (B) Representative PCR genotyping gel of tail DNA. The WT allele is identified by a 224 bp band, while the KO (Δ) allele is identified by a 299 bp band resulting from the deletion. Lanes represent molecular weight marker, homozygous KO, heterozygous (Het), and WT mice. (C) RT-qPCR analysis of *Depp1* mRNA expression in the liver of WT and *Depp1*-KO mice, confirming the complete loss of *Depp1* transcript in the KO group. Data are presented as mean ± SEM.

To investigate the functional role of *Depp1*, we generated Depp1-KO mice using CRISPR/Cas9 (Fig. 4A). Guide RNAs targeting Intron 1 and a region downstream of Exon 2 of the *Depp1* gene were designed to induce double-stranded breaks and subsequent non-homologous end joining repair, resulting in a contiguous deletion encompassing the *Depp1* translational start site and adjacent coding region within Exon 2 (Fig. 4B). This deletion is predicted to disrupt production of full-length DEPP1 protein. qRT-PCR confirmed marked loss of *Depp1* mRNA expression in the Depp1-KO mice (Fig. 4C). Importantly, the qRT-PCR primer pair amplifies a region spanning the Exon 2–Exon 1 transcript junction that overlaps the deleted coding region. Therefore, intact *Depp1* transcript would not be detected in Depp1-KO mice, including under conditions such as CR that normally induce *Depp1* expression in WT mice.

**Figure 5:**
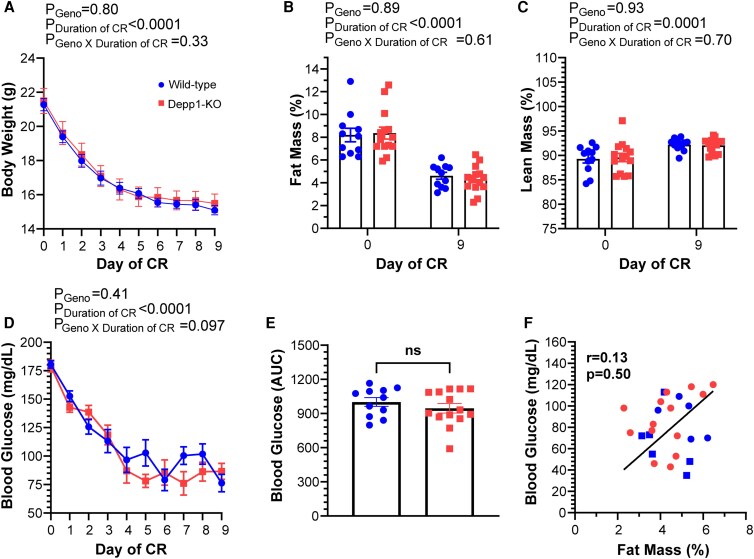
Physiological effects of calorie restriction in WT and Depp1-KO mice. (A) Body weight (g) of WT (blue circles) and Depp1-KO (red squares) mice during a 9-day 60% CR period. (B) Fat mass (%) in WT and Depp1-KO mice at baseline (Day 0) and after 9 days of CR. (C) Lean mass (%) in WT and Depp1-KO mice at baseline (Day 0) and after 9 days of CR. (D) Blood glucose levels (mg/dL) in WT and Depp1-KO mice during the 9-day CR period. (E) AUC for blood glucose during the 9-day CR period in both genotypes. (F) Correlation between blood glucose levels (mg/dL) and fat mass (%) at Day 9 of CR in WT and Depp1-KO mice. Statistical significance was determined by 2-way ANOVA for A-D and by Student *t*-test for panel E (ns: not significant). Pearson correlation coefficient (*r*) and *P*-value are shown in F.

Upon establishing the new Depp1-KO line, male Depp1-KO and WT littermates were submitted to a 9-day version of the CR protocol. Mice of both genotypes exhibited comparable changes in body weight, adiposity, and percent lean mass during CR (Fig. 5A-5C). Similarly, the progressive fall in blood glucose over the course of the CR protocol, which previously has been shown to be exaggerated upon disruption of ghrelin signaling (17, 55-57), was similar in WT and Depp1-KO mice (Fig. 5D and 5E). No correlation was observed between blood glucose and fat mass on Day 9 (Fig. 5F). These data suggest that *Depp1* is not essential for the typical metabolic adaptations to the 9-day CR protocol.

### *Depp1* deletion exacerbates diet-induced obesity and impairs glucose tolerance

**Figure 6:**
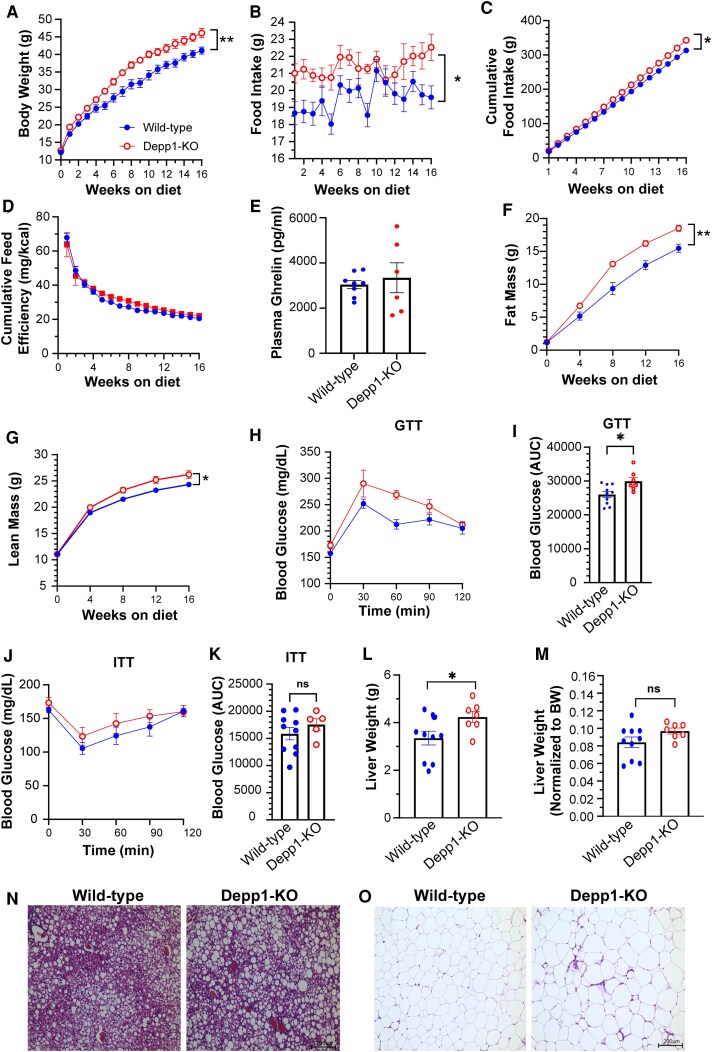
Metabolic phenotyping of *Depp1*-KO male mice on a high-fat diet. (A) Body weight (g) of WT (blue closed circles) and *Depp1*-KO (red open circles) male mice over 16 weeks of HFD feeding. (B) Food intake (g) measured weekly. (C) Cumulative food intake (g) over the 16-week period. (D) Feed efficiency, calculated as body weight gain (g) divided by cumulative food intake (g), over the initial weeks of HFD feeding. (E) Plasma ghrelin concentrations measured by ELISA after 16 weeks of HFD feeding, reflecting circulating active ghrelin levels. (F) Fat mass (g) measured by Echo-MRI. (G) Lean mass (g) measured by Echo-MRI. (H) Blood glucose levels (mg/dL) during an oral GTT performed after 16 weeks of HFD. (I) AUC for the GTT. (J) Blood glucose levels (mg/dL) during an ITT performed after 16 weeks of HFD. (K) AUC for the ITT. (L) Absolute Liver weight (g) at sacrifice. (M) Liver weight normalized to body weight (BW) at sacrifice. (N) Representative H&E stained histological image of the liver from a WT and a Depp1-KO mouse (scale bar: 200 μm). (O) Representative H&E stained histological image of eWAT from a WT and a Depp1-KO mouse. Data are presented as mean ± SEM. Significance was determined by Student *t*-test or 2-way ANOVA for A, B, D, H, and J, followed by Tukey multiple comparisons test when appropriate. Unpaired 2-tailed Student *t*-test was used for C, E, F, G, I, K, L, and M. Significance levels: *P < .05, **P < .01; ns, not significant.

To examine the role of DEPP1 in the setting of DIO, 4-week-old male WT and Depp1-KO mice were fed HFD for 16 weeks, during which time several metabolic parameters were followed. Starting after 8 weeks on the HFD, Depp1-KO mice exhibited a statistically significant higher body weight than WT littermate controls, reaching a 13.8% increase by week 16 (Fig. 6A). Average weekly food intake was increased 9.4% and cumulative food intake was elevated by 9.8%, while cumulative feed efficiency was comparable in Depp1-KO mice vs WT mice (Fig. 6B-6D), suggesting that the exaggerated obesity phenotype in Depp1-KO mice is driven primarily by hyperphagia and not decreased energy expenditure. Furthermore, no significant difference in circulating ghrelin concentrations between WT and Depp1-KO mice were observed under HFD conditions (Fig. 6E). Thus, hyperghrelinemia does not account for the hyperphagic phenotype of the Depp1-KO mice. Nonetheless, it is noteworthy that the ghrelin levels of the Depp1-KO mice are relatively higher than might be expected given their increased body weight [and adiposity (see below)]—a state typically associated with reduced plasma ghrelin (58).

Body composition analysis revealed greater fat mass in Depp1-KO mice from week 8 onward, with a pronounced difference at week 16 (19.6% higher; Fig. 6F). Lean mass was also modestly higher in KO mice at week 16 (by 6.7%; Fig. 6G). After 16 weeks of HFD, Depp1-KO mice displayed impaired glucose tolerance, as evidenced by elevated glucose levels at all time points during oral GTT and a 15.0% increase in AUC (Fig. 6H and 6I), although it remains unclear whether this worsened glucose intolerance is secondary to the exaggerated obesity or occurs independently of it. In contrast, insulin sensitivity, as assessed by ITT, was not significantly altered in Depp1-KO mice (Fig. 6J and 6K). Absolute liver weight measured after 16 weeks of HFD was significantly higher in Depp1-KO mice (Fig. 6L), though liver-to-body weight ratios were without significant differences (Fig. 6M). Histological analyses revealed increased hepatic steatosis and lipid accumulation in Depp1-KO mice (Fig. 6N), as well as enlarged adipocytes in eWAT (Fig. 6O). Collectively, these findings in male mice indicate that loss of *Depp1* exacerbates DIO and impairs glucose homeostasis, suggesting a protective metabolic role for DEPP1 under obesogenic conditions.

Similar HFD feeding studies were performed in female Depp1-KO and WT littermates. In contrast to the phenotype observed in males, female Depp1-KO mice fed HFD showed no significant differences in body weight or other metabolic measures compared with WT controls [Fig. S4A-S4K (43)].

### *Depp1* deletion modulates hepatic transcriptional responses in a gene- and nutritional state–dependent manner

**Figure 7:**
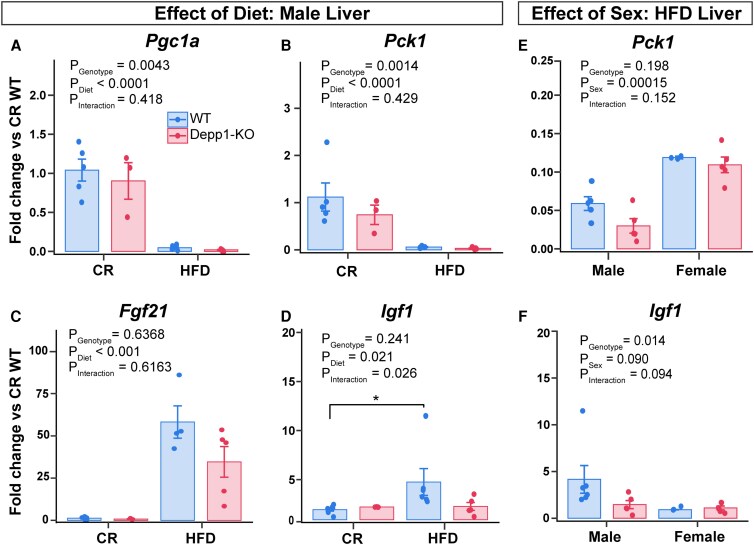
*Depp1* deletion modulates hepatic transcriptional responses in a gene-dependent and nutritional state–dependent manner. Relative mRNA expression of (A) *Pgc1a*, (B) *Pck1*, (C) *Fgf21*, and (D) *Igf1* in the liver of WT and Depp1-KO mice under CR and HFD conditions (male mice). (E, F) Expression of (E) *Pck1* and (F) *Igf1* in male and female mice under HFD conditions. Gene expression was normalized to *B2m* and plotted as fold change relative to CR WT (2^−ΔΔCt^). Data were analyzed using 2-way ANOVA on ΔCt values. *P*-values for main effects (genotype, condition, or sex) and interaction terms are indicated within each panel. Where applicable, significant pairwise comparisons using Tukey test are shown (*P < .05).

To identify molecular correlates of the metabolic phenotypes observed in male Depp1-KO mice because of chronic HFD (exaggerated DIO and glucose intolerance) vs female Depp1-KO mice (no exaggerated DIO or glucose intolerance), we examined effects of feeding condition and genotype on hepatic expression of key metabolic genes. In male WT and Depp1-KO mice, there was a significant effect of feeding condition across all genes analyzed (Fig. 7A-7D). Specifically, expression of *Pgc1a* [*Peroxisome proliferator-activated receptor gamma coactivator 1-α;* a key factor in mitochondrial biogenesis and oxidative metabolism (59)] and *Pck1* [*Phosphoenolpyruvate carboxykinase*; the rate-limiting enzyme in gluconeogenesis (60)] were both strongly suppressed under HFD relative to CR (Fig. 7A and 7B). In addition, genotype-dependent effects were observed for *Pgc1a* and *Pck1*, with expression reduced in Depp1-KO mice [by 13.3% under CR and 55.9% under HFD for *Pgc1a*; by 33.7% under CR and 49.3% under HFD for *Pck1* (Fig. 7A and 7B). These findings suggest that DEPP1 loss is associated with a lower hepatic expression setpoint for selected metabolic genes. However, the absence of any significant genotype-by-condition interactions for *Pgc1a* or *Pck1* suggests that DEPP1 loss does not disrupt the directionality of hepatic transcriptional adaptation to nutritional state.

In contrast, *Fgf21* [*Fibroblast growth factor 21*; a fasting- and stress-induced metabolic hormone that enhances energy expenditure, insulin sensitivity, and adaptive fuel utilization (61)], *Igf1* [*insulin-like growth factor;* an anabolic growth factor that mediates growth hormone actions to promote somatic growth, tissue development, and metabolic homeostasis (62)], and *Leap2* [*Liver-Expressed Antimicrobial Peptide 2*; the endogenous antagonist of GHSR (the ghrelin receptor) (63)] were upregulated (*P* = .071 for *Leap2*) under HFD relative to CR in male WT and Depp1-KO mice [Fig. 7C and 7D; Fig. S5A (43)]. There was no main effect of genotype on *Fgf21*, *Igf1*, or *Leap2*. However, *Igf1* showed a significant genotype-by-feeding condition interaction, driven by attenuation of the HFD-associated increase in *Igf1* expression in male *Depp1*-KO mice (Fig. 7D). Altogether, these data in male Depp1-KO and WT littermates under both CR and DIO conditions suggest that *Depp1* deletion does not modulate the hepatic transcriptional response uniformly across all metabolically relevant genes. Instead, *Depp1* deletion influences expression of certain genes irrespective of feeding conditions (*Pgc1a*, *Pck1*), other genes in a feeding-condition-dependent manner (*Igf1*), and other genes not at all (*Fgf21*, *Leap2*).

Finally, given the sex-dependent metabolic phenotypes observed for Depp1-KO mice under HFD, we assessed the effects of genotype and sex on hepatic transcriptional responses in mice fed HFD. There was a significant effect of sex on *Pck1* expression, with expression 152.3% higher in females than in males, and a trend for an effect of sex on *Igf1* expression (*P* = .090; Fig. 7E and 7F), with expression 78.0% and 24.2% lower in female WT and Depp1-KO mice, respectively. No significant effect of sex on *Leap2*, *Pgc1a*, and *Fgf21* was observed [Fig. S5B-S5D (43)]. There also was a statistical trend for a genotype-by-sex interaction for *Igf1*, such that the genotype-associated decreases in *Igf1* and *Fgf21* observed in male HFD-fed Depp1-KO mice were not evident in females [*Igf1*: *P* = .094, Fig. 7F; *Fgf21*: *P* = .076; Fig. S5D (43)]. These sex-dependent differences in hepatic gene expression may contribute to—or at the very least reflect—the differing metabolic responses to *Depp1* loss observed in males vs females.

## Discussion

Given the contrasting physiological consequences of obesity and CR, it is reasonable to hypothesize that these conditions elicit distinct transcriptional adaptations in key metabolic organs such as the stomach. Although temporally dynamic transcriptional responses to dietary restriction have been characterized in other tissues, stomach-specific data remain limited (64). Here, bulk RNA-seq analysis revealed that CR profoundly remodels the gastric mucosal transcriptome, with enrichment of genes involved in peptide transport, secretion, and extracellular matrix organization, alongside suppression of immune and inflammatory pathways. In contrast, DIO induced a robust proinflammatory transcriptional signature, with minimal overlap in DEGs between CR and DIO conditions. Collectively, these findings support the concept that the gastric mucosa undergoes distinct, nutritional state-dependent transcriptional remodeling in response to opposing metabolic challenges.

A key discovery of this study is the identification of *Depp1* as a CR-responsive gene—not only within the gastric mucosa, but also within several other tissues. Within the gastric mucosa, single-cell transcriptomic analysis and FACS-based validation in *Ghrl^Cre^;tdTomato* reporter mice demonstrated enrichment of *Depp1* in ghrelin cells and selective induction of *Depp1* in ghrelin-positive cells during CR. These findings place *Depp1* within a gastric endocrine population known to respond dynamically to energy deficit and excess. Despite this association with ghrelin cells, whole-body *Depp1* deletion did not impair the major metabolic adaptations to a 9-day CR protocol, including body weight loss, changes in body composition, and the progressive decline in blood glucose. Thus, DEPP1 is strongly induced during CR but is not required for the short-term systemic adaptation to this CR paradigm.

In contrast, *Depp1* deletion exacerbated the metabolic consequences of chronic HFD feeding in male mice. Male *Depp1*-KO mice exhibited greater weight gain, hyperphagia, increased adiposity, hepatic steatosis, and impaired glucose tolerance compared with WT littermates. These findings suggest that DEPP1 plays a protective role under conditions of nutrient excess rather than being essential for the acute metabolic response to energy deficit. The increase in food intake, together with unchanged feed efficiency, suggests that hyperphagia is a major contributor to the exaggerated DIO phenotype. However, because this study did not directly measure energy expenditure, thermogenesis, locomotor activity, meal patterning, or nutrient absorption, additional metabolic cage studies will be required to fully define the mechanisms underlying increased weight gain in *Depp1*-KO mice.

Although *Depp1* was enriched in ghrelin cells and regulated by CR, the DIO phenotype in male *Depp1*-KO mice does not appear to be explained by overt hyperghrelinemia. Indeed, plasma ghrelin levels did not differ significantly between WT and *Depp1*-KO mice after chronic HFD feeding. However, because circulating ghrelin levels typically decline with increasing body weight and obesity, the failure of ghrelin levels to decrease in the more obese Depp1-KO mice suggests a state of relative hyperghrelinemia that could potentially contribute to their exaggerated DIO phenotype through persistent orexigenic signaling. This raises the possibility of subtle alterations in ghrelin cell regulation as a result of Depp1 deletion.

Our findings in Depp1-KO mice provide physiological context for the emerging mechanistic understanding of DEPP1 function. DEPP1 has been identified as a FOXO3 target induced by nutrient deprivation and cellular stress (65), and has been shown to localize to peroxisomes and mitochondria, where it modulates redox signaling, autophagy, apoptosis, mitophagy, and pexophagy (66, 67). Collectively, these studies support a model in which DEPP1 functions as a stress-responsive regulator of organelle remodeling and cellular adaptation. Within this framework, the exaggerated susceptibility of *Depp1*-KO mice to chronic HFD-induced metabolic dysfunction is consistent with a role for DEPP1 in promoting metabolic resilience during sustained nutrient excess or cellular stress. Notably, the absence of a marked baseline metabolic phenotype and the preservation of short-term adaptation to CR suggest that the systemic effects of DEPP1 become most apparent under prolonged metabolic challenge—particularly, long-term HFD exposure.

Our results differ from a previous report in which *Depp1* deletion protected mice from DIO through SIRT1–PPARγ-mediated adipose thermogenesis (68). These differences may reflect variations in the extent of the *Depp1* gene that was deleted, methodological differences in gene targeting (eg, 5-nucleotide deletion in Exon 2 using TALEN gene editing in the prior study vs partial Intron 1 plus complete Exon 2 excision here) and/or distinct physiological pressures imposed by the dietary models [eg, 60% HFD in the prior study, which induces rapid inflammation and strong thermogenic demand, vs 42% “Western-style” HFD here, which could be considered a less severe challenge (69)]. Nonetheless, our findings align with gain-of-function studies in which virally mediated hepatic DEPP1 overexpression ameliorated obesity in *db/db* mice (70), supporting a model in which DEPP1 functions as a negative regulator of weight gain under some metabolic conditions. Resolving these model-dependent differences will require direct comparison of alleles and tissue-specific manipulation of *Depp1* under standardized dietary conditions.

Our study also highlights tissue-specific dependence of *Depp1* regulation on ghrelin. Although CR robustly upregulated *Depp1* across multiple tissues, this response depended on endogenous ghrelin in a tissue-specific manner. In the liver, CR-induced *Depp1* upregulation was absent in GKO mice, suggesting that hepatic *Depp1* induction during CR requires ghrelin signaling. In contrast, CR-induced *Depp1* expression in the gastric mucosa, pancreas, and kidney remained intact in GKO mice, consistent with regulation by ghrelin-independent energy-deficit pathways in these tissues. Notably, acute ghrelin administration to ad libitum–fed mice failed to induce *Depp1* expression in either liver or gastric mucosa, indicating that ghrelin exposure alone is insufficient to recapitulate the CR response. Together, these findings suggest that hepatic *Depp1* induction requires ghrelin signaling within the broader endocrine and metabolic milieu of CR rather than as a direct consequence of acute ghrelin exposure alone.

Hepatic transcriptional profiling further suggested that DEPP1 loss modulates selected metabolic and endocrine pathways rather than broadly disrupting hepatic adaptation to nutritional state. In male mice, HFD strongly suppressed *Pgc1a* and *Pck1* relative to CR, and both genes were modestly reduced in *Depp1*-KO mice across feeding conditions. In contrast, *Igf1* showed a genotype-by-feeding condition interaction, driven by loss of the HFD-associated increase observed in WT mice, whereas *Fgf21* and *Leap2* showed little or no genotype-dependent regulation. These gene-specific effects suggest that DEPP1 loss alters selected components of hepatic metabolic gene expression rather than producing a uniform defect in gluconeogenic or fasting-responsive transcriptional programs. Because these measurements were made at the transcript level, future studies should determine whether these changes correspond to altered protein abundance, hepatic glucose production, growth hormone signaling, and/or hepatokine secretion.

Another important finding was the sex-dependent nature of the exaggerated DIO phenotype resulting from *Depp1* deletion. Male *Depp1*-KO mice developed exaggerated obesity and glucose intolerance during HFD feeding, whereas female *Depp1*-KO mice did not exhibit a similarly exaggerated metabolic phenotype relative to WT controls. Consistent with this divergence, HFD-fed male and female mice exhibited distinct hepatic transcriptional profiles of select genes, including higher *Pck1* expression and lower *Igf1* expression in females, as well as trends toward genotype-by-sex interactions for *Igf1* and *Fgf21*. Together, these findings suggest that sex-dependent hepatic responses may contribute to—or reflect—differential susceptibility to the metabolic effects of *Depp1* loss. However, the present study was not designed to define the mechanisms underlying this sex specificity. Future studies should investigate whether factors such as sex hormones, adipose distribution, hepatic lipid handling, or sex-specific growth hormone signaling modulate the metabolic effects of DEPP1 deficiency.

Several limitations of this study should be acknowledged. First, the use of a whole-body KO model precludes definitive assignment of the DIO phenotype to a specific tissue or cell type. Given the induction of *Depp1* in multiple organs, tissue-specific deletion approaches, including ghrelin cell-specific and hepatocyte-specific models, will be important for defining cell-autonomous functions of DEPP1. Second, circulating ghrelin was assessed only at a single endpoint, and local ghrelin peptide content, post-translational modification, secretion dynamics, and GHSR activity were not assessed. Third, because energy expenditure and substrate utilization were not directly measured, the relative contributions of hyperphagia, thermogenesis, and energy utilization to the obesity phenotype remain unclear. Finally, hepatic transcriptional analyses were restricted to selected candidate genes, and broader transcriptomic, proteomic, and functional analyses will be needed to fully define the pathways linking DEPP1 to hepatic metabolism.

In summary, this study identifies DEPP1 as a nutrient-responsive gene enriched in gastric ghrelin cells and regulated by CR across multiple tissues. Although DEPP1 is dispensable for major short-term metabolic adaptations to CR, it appears to protect against the adverse metabolic consequences of chronic HFD feeding in male mice. Together with prior evidence implicating DEPP1 in stress-responsive organelle remodeling, these findings support a model in which DEPP1 promotes metabolic resilience during sustained nutrient excess. Future studies using tissue-specific models and integrated metabolic phenotyping will be needed to define how DEPP1 acts across gastric endocrine, hepatic, and adipose pathways to regulate systemic energy balance.

## Data Availability

Original data generated and analyzed during this study are included in this published article or in the data repositories listed in references, including the NCBI Gene Expression Omnibus under accession number GSE315851 (71) and the Texas Data Repository (43).

## Abbreviations

CR: calorie restriction
DIO: diet-induced obesity
GI: gastrointestinal
GMC: gastric mucosal cell
GKO: Ghrelin-knockout
GO: gene ontology
KO: knockout
sgRNA: short-guide RNA
WT: wild type
